# Characterization and Genome Analysis of *Mycocentrospora acerina*, the Causal Agent of *Panax notoginseng* Round Spot Disease in China

**DOI:** 10.3390/jof11110811

**Published:** 2025-11-15

**Authors:** Kuan Yang, Yinglong Deng, Xiang Li, Chao Li, Xiahong He, Liwei Guo

**Affiliations:** 1 Henan Province Key Laboratory of Zhang Zhongjing Formulae and Herbs for Immunoregulation, Nanyang Institute of Technology, Nanyang 473306, China; ykweter@outlook.com (K.Y.);; 2 State Key Laboratory for Conservation and Utilization of Bio-Resources in Yunnan, Yunnan Agricultural University, Kunming 650201, China; 3 Key Laboratory for Forest Resources Conservation and Use in the Southwest Mountains of China, Ministry of Education, Southwest Forestry University, Kunming 650224, China

**Keywords:** *Mycocentrospora acerina*, *Panax notoginseng*, genome annotation, CAZyme profiles

## Abstract

The pathogenic fungus *Mycocentrospora acerina*, responsible for *Panax notoginseng* round spot disease, poses a serious threat to the development of the *P. notoginseng* industry. To investigate its genetic information and potential pathogenic mechanisms, this study employed nanopore third-generation sequencing technology to conduct de novo genome sequencing and analysis of *M. acerina*, followed by an assessment of its plant cell wall-degrading enzyme activities. The sequencing results revealed that the *M. acerina* genome has a total length of 37.03 Mb, a GC content of 47.68%, an N50 value of 1.66 Mb, and a repeat sequence proportion of 9.37%. A total of 9989 protein-coding genes were predicted. Genome annotation identified 499 carbohydrate-active enzyme (CAZyme) family genes—more than those found in *Botrytis cinerea* (469), *Phanerochaete chrysosporium* (381), and *Erysiphe necator* (136). Moreover, *M. acerina* harbors a relatively large number of genes encoding plant cell wall-degrading enzymes. Experimental measurements of cell wall-degrading enzyme activities were consistent with the genomic predictions, demonstrating that *M. acerina* exhibits strong abilities to degrade cellulose, pectin, and lignin. This study provides new insights into the pathogenic mechanisms of *M. acerina* and establishes a theoretical foundation for developing potential control strategies for *P. notoginseng* round spot disease.

## 1. Introduction

*Panax notoginseng* (Burk.) F. H. Chen, commonly known as Sanqi, Tianqi, or Jinbuhuan, is a perennial herb belonging to the genus Panax of the family Araliaceae. It is an endemic and highly valued traditional Chinese medicinal herb renowned for its properties of promoting blood circulation, dissipating blood stasis, reducing swelling, and alleviating pain [1,2]. Modern pharmacological studies have revealed that *P. notoginseng* contains a variety of chemical constituents, including saponins, flavonoids, cyclic peptides, sterols, carbohydrates, and amino acids [3,4]. The cultivation of *P. notoginseng* demands stringent environmental conditions—warm winters and cool summers, the absence of extreme cold or heat, partial shade, and a humid climate—and is therefore particularly susceptible to diseases [5]. Among them, *P. notoginseng* round spot disease (PRSD) is the most significant foliar disease encountered during cultivation, first reported in Wenshan Prefecture, Yunnan Province, in 1993 [6].

In the early stage of PRSD, water-soaked, circular lesions appear on the leaves, gradually turning brown over time. Under humid conditions, a white fungal mycelial layer develops on the lesion surface. In the advanced stage, lesions merge, causing leaf decay and abscission (Figure S1). This disease is highly destructive; if not promptly controlled once it occurs, the incidence rate can exceed 90.00% in extreme years, with the potential to devastate entire plantations, leading to severe economic losses for growers [7]. The occurrence of PRSD is predominantly concentrated in the rainy season. Because the pathogen thrives in low-temperature, high-humidity environments and is highly infectious, outbreaks during the rainy season often result in large-scale spread and epidemics. The disease is caused by the ascomycetous fungus *Mycocentrospora acerina* (Hartig) Deighton. This pathogen was first reported in Germany in 1880 infecting leaves of maple trees. It is mainly distributed across Europe and North America, with a wide host range including plants in the families Aceraceae, Brassicaceae, and Solanaceae. Under cool and moist conditions, it can cause diseases in a variety of vegetable and ornamental plants, such as carrots, lettuce, and primroses [8].

Genomics refers to the scientific study of the entire DNA sequence content of an organism’s genome and its sequencing methodologies. It represents one of the most advanced disciplines in the field of life sciences. With the rapid development of sequencing technologies, fungal genomics has progressed significantly, greatly promoting research on fungal physiology and genetic diversity [9,10]. Applying genome sequencing technology to the study of phytopathogenic fungi—sequencing their genomes and using bioinformatics approaches to identify pathogenicity-related genes or signal pathways—has become an effective method for investigating the molecular mechanisms underlying pathogenicity in plant pathogens [11,12,13]. Shang et al. [14] for the first time obtained a chromosome-level reference genome of *Fusarium foetens*, a novel pathogenic fungus causing sweet potato root rot, and identified the key pathogenic effector protein FfRP752 along with its functional target. Bhadauria et al. [15] decoded the complete genomic features of *Epicoccum sorghinum*, the causal agent of leaf sheath blight in maize, and elucidated the regulatory patterns of gene expression during infection through genome and transcriptome analyses. Zhang et al. [16] successfully sequenced the entire genome of Rice Yellow Dwarf Phytoplasma (RYDP) and revealed how this pathogen disrupts the normal development of rice through its effector proteins. PRSD poses a serious threat to the *P. notoginseng* industry. Current research on its pathogenic fungus has mainly concentrated on physiological characteristics and control strategies, while genomic information remains largely unexplored. To investigate the genetic background and molecular pathogenic mechanisms of *M. acerina*, this study employed nanopore third-generation sequencing technology to perform de novo genome sequencing and analysis. Particular emphasis was placed on characterizing the types and numbers of genes encoding carbohydrate-active enzymes (CAZymes), along with further assays of *M. acerina*’s plant cell wall-degrading enzyme activities, aiming to elucidate its potential pathogenic mechanisms.

## 2. Materials and Methods

### 2.1. Isolation, Screening, and Cultivation of M. acerina

In our previous work, a total of 30 isolates of the pathogen causing PRSD were successfully obtained from diseased *P. notoginseng* fields, and the pathogen was identified as *M. acerina* [7]. Measurement of mycelial growth rate of isolated strains. All purified isolates were cultured on PDA plates at 20 °C for 10 days. Discs (5 mm in diameter) were cut from the periphery of each colony and placed mycelium-side down onto fresh PDA plates. The cultures were incubated at 20 °C under continuous light for 7 days. Colony diameters were measured every 24 h, with four replicates for each isolate.

Mycelial growth rate (mm/day) = (average colony diameter − disc diameter)/days of incubation.

Determination of pathogenicity variation among isolates. Fresh, healthy, and uniformly growing one-year-old leaves of *P. notoginseng* were selected. Leaves were surface-sterilized with 75% ethanol for 2 min, rinsed 3–4 times with sterile water, and air-dried in a laminar flow hood. They were then placed into disposable sterile Petri dishes (90 mm) pre-loaded with moist sterile filter paper to maintain humidity, with five leaves per dish. For each isolate, 30 leaves were inoculated, using 10 leaves per replicate. After culturing the test fungal isolates on PDA plates at 20 °C for 10 days, 5 mm-diameter mycelial plugs were excised from the colony margins. These plugs were inverted and inoculated onto the surfaces of *P. notoginseng* leaves (without wounding). The inoculated leaves were then placed in an illuminated incubator set at 20 °C, with alternating light and dark cycles (12 h/12 h) and a relative humidity greater than 90%. After 7 days, disease symptoms were assessed, and the diameters of the lesions were measured with a ruler. The pathogenicity of each isolate was determined based on the size of the lesions. The pathogenicity grading criteria for *M. acerina* isolates were presented in Table 1.

**Table 1:** Pathogenicity grading standard.

Lesion Diameter/cm	Level
0	0
0–0.5	1
0.5–1.0	2
1.0–1.5	3
1.5–2.0	4
>2.0	5

### 2.2. Genome Sequencing, Assembly, and Annotation

After culturing the *M. acerina* strain on PDA medium at 20 °C under a 16 h light/8 h dark photoperiod for 7 days, the resulting mycelium was collected for high-purity genomic DNA extraction. Genomic DNA was isolated using a fungal genomic DNA extraction kit (Genomic-tip 20/G, QIAGEN, Hilden, Germany) following the manufacturer’s protocol. The purity, concentration, and integrity of the extracted DNA were assessed using a Nanodrop spectrophotometer, Qubit fluorometer, and 0.35% agarose gel electrophoresis, respectively. The extracted genomic DNA was considered suitable for library construction if it met the following quality criteria: total yield ≥ 1.5 µg, concentration ≥ 50 ng/µL, OD260/280 ratio between 1.7 and 2.2, and OD260/230 ratio between 1.7 and 2.5.

The whole-genome sequencing of *M. acerina* was commissioned to Beijing Biomarker Technologies Co., Ltd. (Beijing, China), utilizing the Oxford Nanopore PromethION48 system. First, the Flow Cell Priming Mix was prepared using the sequencing chip reagent kit (EXP-FLP001.PRO.6, Nanopore, Oxford, England). Subsequently, the sequencing library was constructed with the ligation sequencing kit (SQK-LSK109, Nanopore, Oxford, England). Sequencing was performed using PromethION Flow Cells (FLO-PRO002, Nanopore) on the PromethION48 sequencer, with the MinKNOW software v2.2 controlling the run, and a default sequencing duration of 72 h.

The raw data generated by Nanopore sequencing were initially stored in the binary fast5 format, which contains all original sequencing signals, with each read corresponding to a single fast5 file. After base calling using the Albacore software v2.3.4 from the MinKNOW package, the data were converted from fast5 to fastq format. Subsequently, reads were further filtered to remove adapters, low-quality sequences, and short fragments (length < 2000 bp), resulting in the final dataset. Genome assembly was performed using the NECAT software v0.01, and the assembled genome was further polished with Pilon [17] using second-generation sequencing data to achieve higher accuracy in the final genome sequence.

Gene structure prediction was primarily performed using ab initio methods and homology-based prediction using protein sequences, and the results from the two approaches were subsequently integrated. Ab initio prediction was performed using Genscan [18], Augustus v2.4 [19], GlimmerHMM v3.0.4 [20], GeneID v1.4 [21], and SNAP (version 2006-07-28) [22]. Homology-based protein prediction was conducted with GeMoMa v1.3.1 [23]. Finally, the predictions from these two methods were integrated using EVM v1.1.1 [24].

The predicted gene sequences were subjected to BLAST searches [25] against functional databases such as KOG, KEGG, Swiss-Prot, TrEMBL, and Nr to obtain gene functional annotations. Based on the BLAST alignment against the Nr database, Blast2GO [26] was employed for Gene Ontology (GO) [27] functional annotation. In addition, hmmer [28] was used to perform Pfam functional annotation [29].

### 2.3. Identification of CAZymes and Secondary Metabolite Gene Clusters

Secondary metabolite biosynthetic gene clusters (BGCs) were predicted using the AntiSMASH online platform (https://antismash.secondarymetabolites.org/) with default parameters [30]. The dbCAN web server (https://bcb.unl.edu/dbCAN2/, accessed on 20 October 2022) was employed to perform genome annotations, utilizing the HMMER program to search against the CAZy database (http://www.cazy.org/) [31]. Results with an E-value < 1 × 10^−5^ and sequence coverage > 0.35 were compiled. This approach was applied to the genomic annotation and analysis of *M. acerina*, *Phanerochaete chrysosporium*, *Botrytis cinerea*, *Pyricularia oryzae*, and *Erysiphe necator*.

The genome datasets used for comparative genomics were obtained from the following sources: *Phanerochaete chrysosporium* strain ATCC 20,696 (https://www.ncbi.nlm.nih.gov/datasets/genome/GCA_001910725.1/, accessed on 10 October 2022); *Botrytis cinerea* strain T4 (https://www.ncbi.nlm.nih.gov/datasets/genome/GCA_000292645.1/, accessed on 10 October 2022); *Pyricularia oryzae* strain MZ5-1-6 (https://www.ncbi.nlm.nih.gov/datasets/genome/GCA_004346965.1/, accessed on 10 October 2022); and *Erysiphe necator strain* c (https://www.ncbi.nlm.nih.gov/datasets/genome/GCA_000798715.1/, accessed on 10 October 2022).

### 2.4. Measurement of Cell Wall-Degrading Enzyme Activity

First, lignin-activating enzyme-inducing medium, pectinase-inducing medium, and cellulase-inducing medium were prepared.

Lignin-activating enzyme-inducing medium: KNO_3_ 2.00 g, KCl 0.50 g, FeSO_4_ 0.01 g, K_2_HPO_4_ 1.00 g, MgSO_4_·7H_2_O 0.50 g, vitamin B_1_ 0.10 g, L-asparaginase 0.50 g, lignin 10 g, and ddH_2_O to a final volume of 1 L.

Pectinase-inducing medium: KNO_3_ 2.00 g, KCl 0.50 g, FeSO_4_ 0.01 g, K_2_HPO_4_ 1.00 g, MgSO_4_·7H_2_O 0.50 g, vitamin B_1_ 0.10 g, L-asparaginase 0.50 g, pectin 10 g, and ddH_2_O to a final volume of 1 L.

Cellulase-inducing medium: KNO_3_ 2.00 g, KCl 0.50 g, FeSO_4_ 0.01 g, K_2_HPO_4_ 1.00 g, MgSO_4_·7H_2_O 0.50 g, vitamin B_1_ 0.10 g, L-asparaginase 0.50 g, cellulose 10 g, and ddH_2_O to a final volume of 1 L.

The prepared media were dispensed into 100 mL Erlenmeyer flasks, each containing 75 mL of medium. After autoclaving at 121 °C for 20 min and cooling to room temperature, pre-cultured *M. acerina*, *P. chrysosporium*, *B. cinerea*, and *P. oryzae* were aseptically inoculated into the respective media under aseptic conditions in a laminar flow cabinet (the *B. cinerea* and *P. oryzae* strains used for cell wall-degrading enzyme activity assays were provided by the Key Laboratory of Plant Pathology, Yunnan Agricultural University; the *P. chrysosporium* strain NDM3-2 was obtained from the China General Microbiological Culture Collection Center, Beijing, China). For each culture, five mycelial plugs (5 mm in diameter, fully overgrown with hyphae) were inoculated, and the flasks were sealed with a gas-permeable, contamination-resistant membrane.

Following inoculation, *M. acerina* was incubated on a shaker at 20 °C (140 rpm), while *P. chrysosporium*, *B. cinerea*, and *P. oryzae* were incubated at 25 °C (140 rpm) under natural light conditions. Samples were collected at 24, 48, and 72 h post-inoculation (hpi) for detection of pectinase and cellulase activities, while lignin-degrading activity was assessed by measuring peroxidase, laccase, and manganese peroxidase activities. All five enzyme activities were determined using commercial assay kits according to the manufacturer’s instructions (Suzhou Keming Biotechnology Co., Ltd., Suzhou, China).

### 2.5. Statistical Analysis and Visualization

The data were analyzed by one-way analysis of variance (ANOVA) followed by Tukey’s post hoc test using SPSS 18.0 software (SPSS Inc., Chicago, IL, USA). Figures were produced using the ggplot2 package in R v4.2.0 and GraphPad Prism 8.3.0.

## 3. Results

### 3.1. Assessment of Mycelial Growth Rate and Pathogenicity of M. acerina Isolates

**Figure 1:**
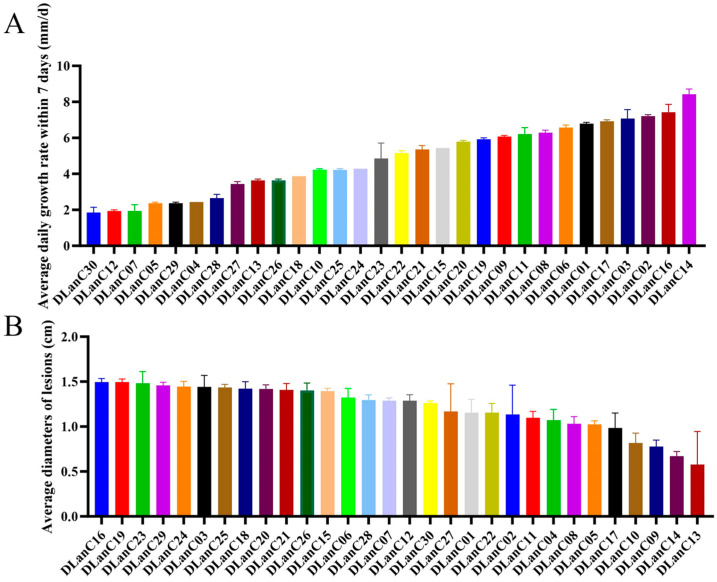
Mycelial growth rate and pathogenicity of 30 isolates of *M. acerina*. (**A**) Mycelial growth rate. (**B**) Diameter of lesions on *Panax notoginseng* caused by *M. acerina* infection.

The mycelial growth rate and pathogenicity of 30 isolates of *M. acerina* obtained in the preliminary separation were determined. The results revealed considerable variation in growth rates among the strains (Figure 1A). The fastest-growing isolate was DlanC14, with an average growth rate of 8.43 mm/day, while the slowest, DlanC30, exhibited a growth rate of 1.86 mm/day. The growth rate of the mycelium is indicative of its vitality. Isolates with an average daily mycelial growth rate exceeding 6 mm were selected for subsequent experiments. These isolates included DLanC09, DLanC11, DLanC08, DLanC06, DLanC01, DLanC17, DLanC03, DLanC02, DLanC16, and DLanC14.

The pathogenicity of different isolated strains was assessed by recording the incidence and measuring lesion diameter on *P. notoginseng* leaves seven days after inoculation. Statistical analysis of disease incidence and lesion size showed that all 30 isolates were capable of infecting *P. notoginseng* leaves, with a 100% infection rate; however, lesion diameters varied among strains (Figure 1B). The isolate DLanC16 exhibited the highest virulence, producing an average lesion diameter of 1.49 cm, whereas DLanC13 showed the weakest virulence, with an average lesion diameter of 0.58 cm. According to the pathogenicity classification standards, five of the thirty pathogenic isolates were categorized as level 2, while the remaining twenty-five were classified as level 3 (Table S1).

Based on the results of mycelial growth rate and pathogenicity assessments, isolate DLanC16 was selected for genome sequencing.

### 3.2. Genome Sequencing, Assembly, and Annotation of Mycocentrospora acerina

Using the Nanopore third-generation sequencing platform, raw data were obtained and subjected to filtering to remove adapters, short fragments, and low-quality reads. A total of 4,741,936,817 bp of clean data were retained, with a sequencing depth of 128.03×. The assembly yielded a complete genome of 37.03 Mb, with a GC content of 47.68%, consisting of 28 contigs and an N50 value of 1.66 Mb. Repetitive sequences accounted for 9.37% of the genome (Table 2). Mapping of second-generation sequencing data indicated a coverage rate of 99.96%, and BUSCO assessment estimated a genome completeness of 98.28%, demonstrating the high integrity of the assembly. Gene prediction results identified a total of 9989 genes, with an average length of 1705.5 bp and a gene density of 270 genes per Mb. Each gene contained an average of 2.8 exons with an average exon length of 542.31 bp, and 1.8 introns per gene with an average intron length of 103.69 bp. The genome assembly was deposited in the NCBI genome database under BioProject ID: PRJNA1347288.

Gene prediction identified a total of 9989 genes. Comparing the predicted gene set against various functional databases yielded the following results (Table S2): 5394 genes (54% of all predicted genes) were annotated in the GO database based on protein sequence annotations; 3340 genes (33.43%) in KEGG; 5461 genes (54.67%) in KOG; 7724 genes (77.32%) in Pfam; 6353 genes (63.60%) in Swiss-Prot; 9871 genes (98.82%) in TrEMBL; and 9867 genes (98.78%) in the nr database.

**Figure 2:**
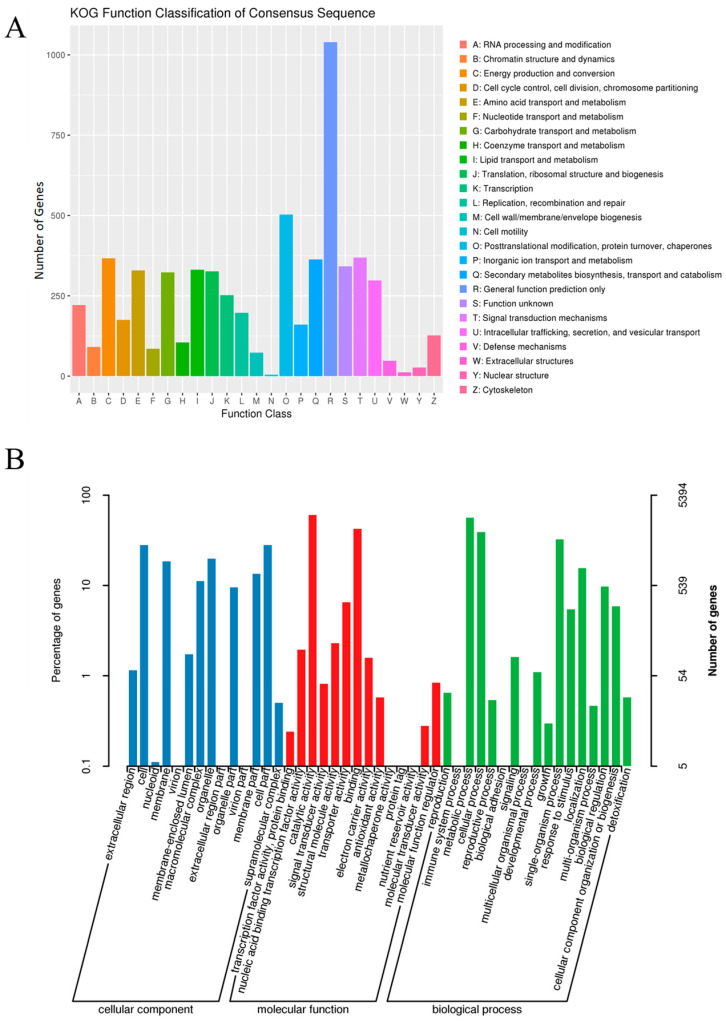
Classification statistics of KOG and GO function annotation results. (**A**) KOG function annotation results. (**B**) GO function annotation Results.

In the KOG database, the top five functional categories with the largest number of annotated genes were posttranslational modification, secondary metabolite biosynthesis, energy production and conversion, signal transduction mechanisms, and lipid transport and metabolism (Figure 2A).

In the GO database, the top five functional categories in terms of annotated gene numbers were as follows. For cellular components, they were cell part, cell, organelle, membrane, and membrane part. For molecular functions, the top categories were catalytic activity, binding, transporter activity, structural molecule activity, and nucleic acid binding transcription factor activity. For biological processes, the dominant categories were metabolic process, cellular process, single-organism process, localization, and biological regulation (Figure 2B).

**Figure 3:**
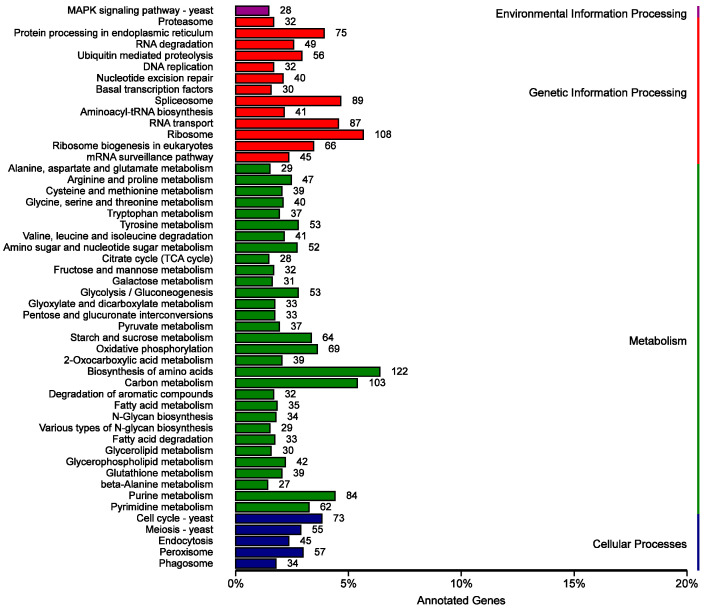
Classification statistics of KEGG annotation results.

In the KEGG database, the top five functional categories with the largest number of annotated genes were biosynthesis of amino acids, carbon metabolism, purine metabolism, oxidative phosphorylation, and starch and sucrose metabolism (Figure 3).

### 3.3. Comparative Genomic Analysis

#### 3.3.1. Comparison of Basic Genomic Features

For comparative genomic analysis with *M. acerina*, representative fungi from different nutritional modes were selected: the saprotrophic white-rot model fungus *P. chrysosporium* (notable for its strong lignin-degrading capacity), the necrotrophic model fungus *B. cinerea*, the hemi-biotrophic model fungus *P. oryzae*, and the biotrophic fungus *E. necator*. A comparison of basic genomic features revealed that *M. acerina* possessed the smallest genome at 37.04 Mb, followed in ascending order by *P. chrysosporium* (39.21 Mb), *B. cinerea* (39.50 Mb), *P. oryzae* (42.70 Mb), and the largest genome in *E. necator* at 52.51 Mb (Table 3). In terms of protein-coding genes, *B. cinerea* had the highest number, with 16,360 predicted genes, followed by *P. oryzae*. *E. necator* exhibited the lowest count, with a total of 6484 predicted protein-coding genes.

#### 3.3.2. Analysis of Genes Related to Secondary Metabolism

Secondary metabolites play a critical role in the ability of fungi to colonize specific hosts and tissues. Plant pathogenic fungi can produce diverse classes of toxic secondary metabolites, including polyketides, peptides, terpenes, and indole alkaloids, which contribute to the death of host cells. The biosynthesis of these secondary metabolites predominantly relies on four key enzymes: polyketide synthases (PKS), nonribosomal peptide synthases (NRPS), terpene synthases (TS), and dimethylallyl transferases (DMAT) [36,37,38].

A statistical analysis was conducted on the number and types of core genes associated with the synthesis of secondary metabolites in five fungal species (Table S3). The results showed that *P. oryzae* possessed the highest number of core genes, with 56 in total, followed by *B. cinerea* and *M. acerina*, with 44 and 42 genes respectively. *E. necator* had the fewest, with only 5. Notably, the saprophytic fungus *P. chrysosporium* contained only 2 PKS genes, whereas the plant pathogenic fungus *P. oryzae* had the largest number, with 23 PKS genes. *M. acerina* ranked second with 16, and *B. cinerea* had 14 PKS genes. Overall, *M. acerina* contains a relatively high number of core genes related to secondary metabolite synthesis, with its quantity and diversity being similar to those of *B. cinerea*.

#### 3.3.3. Carbohydrate-Active Enzymes (CAZymes)

CAZymes are a family of proteins with diverse catalytic activities on carbohydrates and are closely associated with fungal growth, development, and pathogenicity. Functionally, CAZymes are classified into glycoside hydrolases (GHs), glycosyltransferases (GTs), polysaccharide lyases (PLs), carbohydrate esterases (CEs), auxiliary activities (AAs), and carbohydrate-binding modules (CBMs) [39]. In this study, the types and numbers of CAZyme coding genes in *P. oryzae*, *P. chrysosporium*, *B. cinerea*, and *E. necator* were compared with those in *M. acerina* (Table 4). The results revealed that *P. oryzae* possessed the highest number of CAZyme coding genes (544), followed by *M. acerina* (499), *B. cinerea* (469), and *P. chrysosporium* (381). In contrast, the obligate biotroph *E. necator* displayed the lowest count, with only 136 genes.

The plant cell wall serves as a critical interface for interactions between hosts and pathogenic fungi, functioning as a primary barrier during the invasion process. It is mainly composed of polymers such as cellulose, hemicellulose, pectin, and lignin. During the host–pathogen recognition phase, pathogenic fungi secrete cell wall-degrading enzymes that break down the host’s cell wall and cuticle, thereby facilitating pathogen penetration, colonization, and spread [40]. CAZymes include a class of plant cell wall-degrading enzymes (PCWDEs), which are crucial for the successful invasion and infection of host plants by fungal pathogens. Based on the type of substrate targeted, PCWDEs can be further classified into cellulases, hemicellulases, ligninases, and pectinases. Cellulases are primarily grouped within GH (1, 3, 6, 7, 12, 45), AA9, and CBM1 families; hemicellulases mainly belong to CE (1, 2, 3, 5, 12, 15, 16) and GH (10, 11, 26, 27, 31, 35, 36, 43, 53, 54, 93) families; ligninases are largely classified under AA (1, 2, 3, 4, 5, 6, 7, 8) families; and pectinases are predominantly assigned to GH (28, 78, 95, 105), PL (1, 3, 10), and CE8 families [41,42].

**Figure 4:**
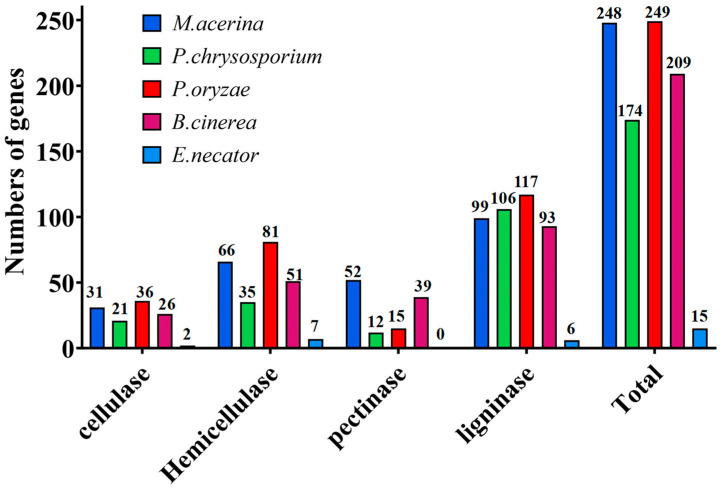
Comparison of the number of plant cell wall-degrading enzyme-encoding genes in five fungal species.

**Figure 5:**
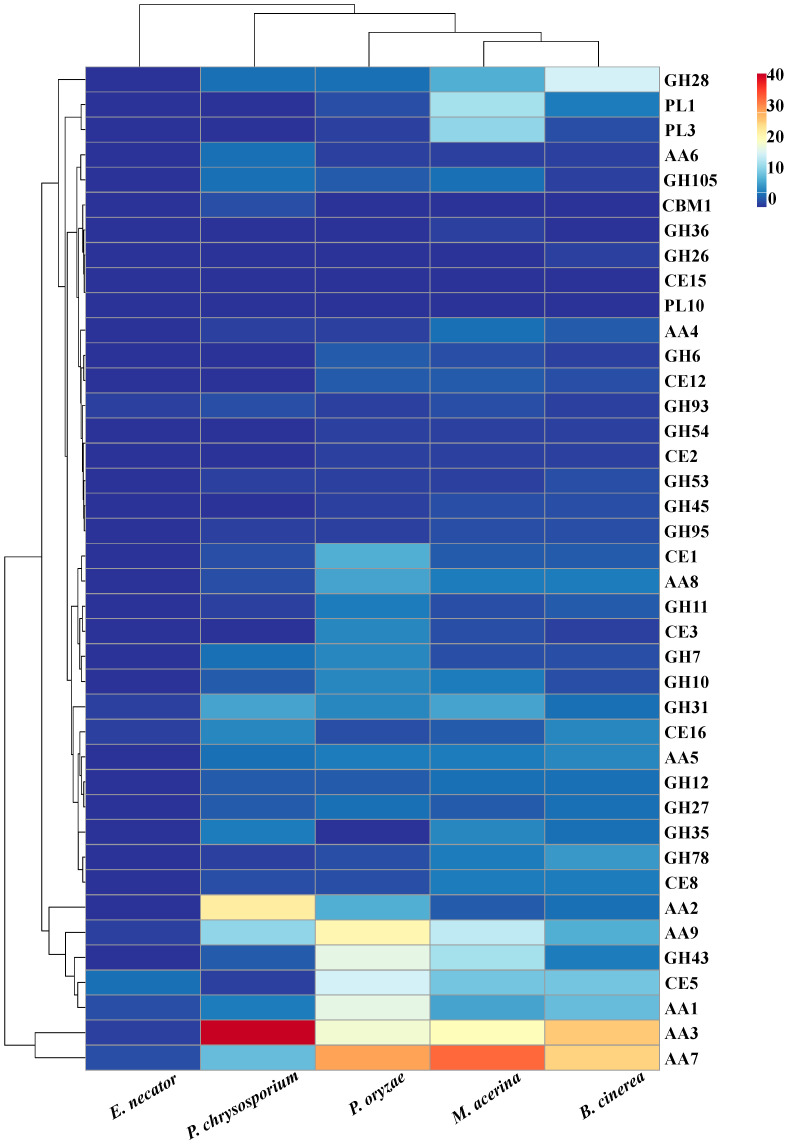
Cluster analysis of the types and quantities of plant cell wall-degrading enzyme-encoding genes in five fungal species.

A comparative analysis of the number of genes encoding PCWDEs in five fungal species was conducted (Figure 4). Among cellulases, *P. oryzae* possessed the largest number of encoding genes, followed by *M. acerina*, while *E. necator* had the fewest, with only two genes. For hemicellulases, *P. oryzae* again exhibited the greatest number of encoding genes (81), followed by *M. acerina* (66), *B. cinerea* (51), *P. chrysosporium* (35), and *E. necator* (7). Regarding pectinases, *M. acerina* had the highest number of encoding genes (52), followed by *B. cinerea* (39), whereas *P. oryzae* and *P. chrysosporium* contained relatively fewer, with 15 and 12 genes, respectively. In terms of lignin-degrading enzymes, *P. oryzae* had the largest number of encoding genes (117), followed by *P. chrysosporium* (106), *M. acerina* (99), *B. cinerea* (93), and *E. necator* (7). When considering the total number of genes encoding enzymes for the degradation of various plant cell wall components, *P. oryzae* remained the species with the highest total (249), closely followed by *M. acerina* (248), with only a minimal difference between them. *B. cinerea* possessed 209 such genes, *P. chrysosporium* 174, and *E. necator* the fewest, with only 15 in total. Further clustering analysis of these PCWDEs revealed that *M. acerina* and *B. cinerea* grouped together in a distinct clade (Figure 5), indicating a close phylogenetic relationship and a high degree of similarity in their enzyme profiles. Notably, *M. acerina* possesses a greater number of genes encoding enzymes belonging to the AA3, PL1, and PL3 families.

### 3.4. Determination of Lignin Degradation Capacity

**Figure 6:**
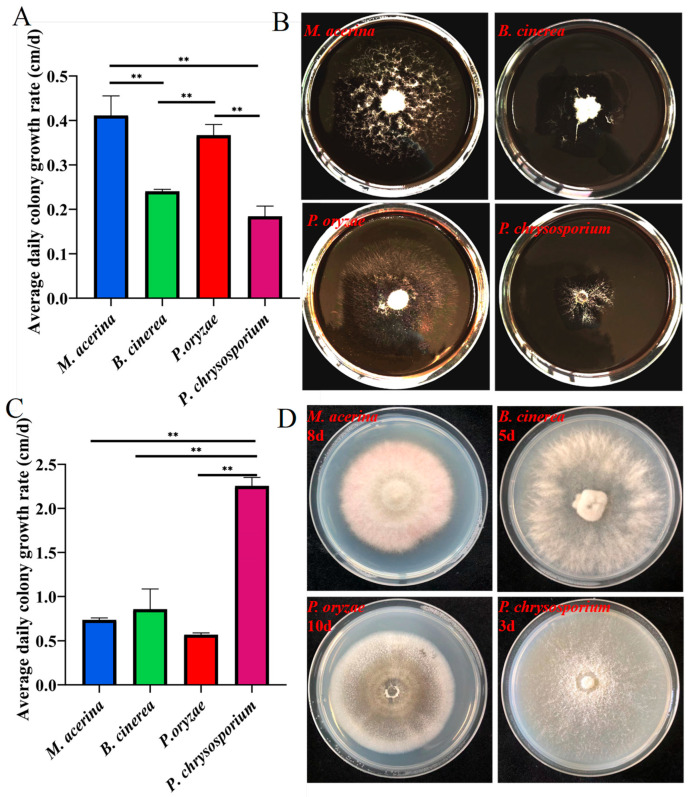
Determination of the lignin utilization capacity of four fungal species. (**A**,**B**) Growth rate and morphological characteristics of four fungal species on agar medium containing 1.6% lignin (** *p* < 0.01, n = 3). (**C**,**D**) Growth rates and colony sizes of four fungal species on PDA medium (** *p* < 0.01, n = 3).

A lignin-based medium, with lignin as the sole carbon source, was prepared to cultivate *M. acerina*, *P. oryzae*, *P. chrysosporium*, and *B. cinerea*. Measurement of colony diameters began once the colony of any strain exceeded two-thirds of the Petri dish diameter. After 18 days, *M. acerina* was the first to surpass two-thirds of the dish, exhibiting the fastest growth rate. The results indicated that all four fungi were capable of utilizing lignin as the sole carbon source for growth (Figure 6B). Among them, *M. acerina* displayed the highest average daily growth rate at 0.41 cm/day, followed by *P. oryzae* at 0.37 cm/day, *B. cinerea* at 0.24 cm/day, and *P. chrysosporium* at 0.18 cm/day, the slowest. There was no statistically significant difference in growth rate between *M. acerina* and *P. oryzae*, nor between *P. chrysosporium* and *B. cinerea*. The growth rates of *M. acerina* and *P. oryzae* were significantly higher than those of *P. chrysosporium* and *B. cinerea* (Figure 6A). Notably, *P. chrysosporium*, while showing slow growth on lignin-based medium, grew rapidly on PDA medium (Figure 6C,D), achieving significantly higher growth rates than the other three pathogens, thereby demonstrating strong vitality and efficient nutrient utilization capacity.

### 3.5. Determination of Cell Wall-Degrading Enzyme Activity

**Figure 7:**
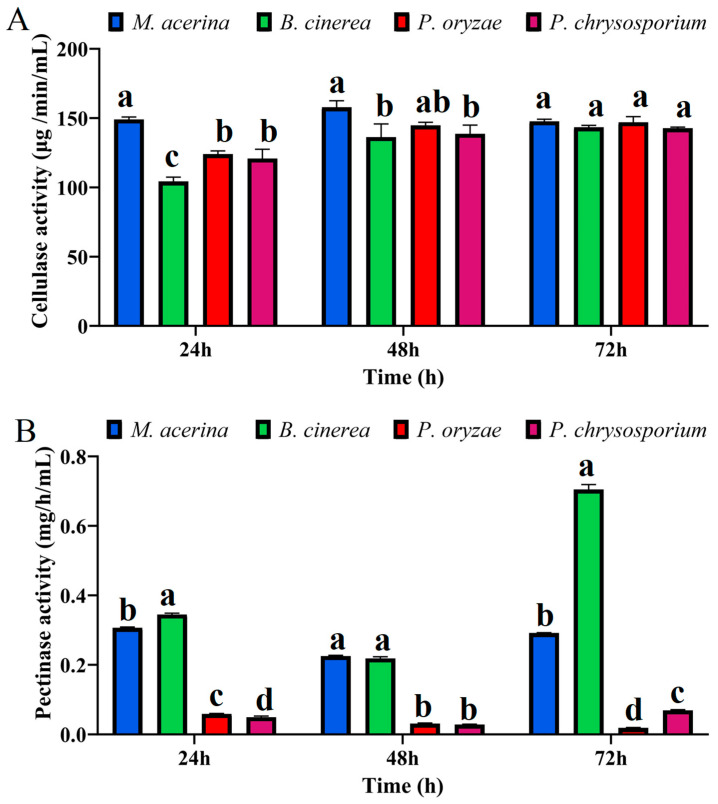
Cellulase and pectinase activities of four fungal species at different culture time points. (**A**) Cellulase activities. (**B**) Pectinase activities. Different letters above the plots indicate significant differences between treatments (*p* < 0.05, n = 3).

To further verify the plant cell wall-degrading capacity of *M. acerina*, we assayed the activities of cellulases, pectinases, and lignin-degrading enzymes and compared them with those of *P. oryzae*, *B. cinerea*, and *P. chrysosporium*. The lignin-degrading enzyme assays included laccase, manganese peroxidase, and lignin peroxidase. For cellulase (Figure 7A), at 24 hpi, *M. acerina* exhibited significantly higher enzyme activity than the other three fungal species, while *B. cinerea* showed the lowest activity. At 48 hpi, *M. acerina* still displayed the highest cellulase activity, followed by *P. oryzae*; the activities of *M. acerina* and *P. oryzae* did not differ significantly from each other but were significantly higher than those of *B. cinerea* and *P. chrysosporium*. By 72 hpi, no significant differences in enzyme activity were observed among the four species.

For pectinase activity (Figure 7B), at 24 hpi, *B. cinerea* exhibited the highest activity, significantly exceeding the other three fungi. *M. acerina* ranked second, with activity significantly higher than that of *P. oryzae* and *P. chrysosporium*. *P. oryzae* and *P. chrysosporium* showed relatively low activities, with a large gap compared with *B. cinerea* and *M. acerina*. At 48 hpi, *M. acerina* and *B. cinerea* again displayed the highest activities, with no significant difference between them, but both were significantly higher than *P. oryzae* and *P. chrysosporium*. By 72 hpi, *B. cinerea* still had the highest activity, significantly higher than the other three, followed by *M. acerina*, which was significantly higher than *P. oryzae* and *P. chrysosporium*; the differences remained substantial. Overall, these pectinase activity patterns are consistent with prior predictions based on the numbers of pectinase-encoding genes in the four fungi.

**Figure 8:**
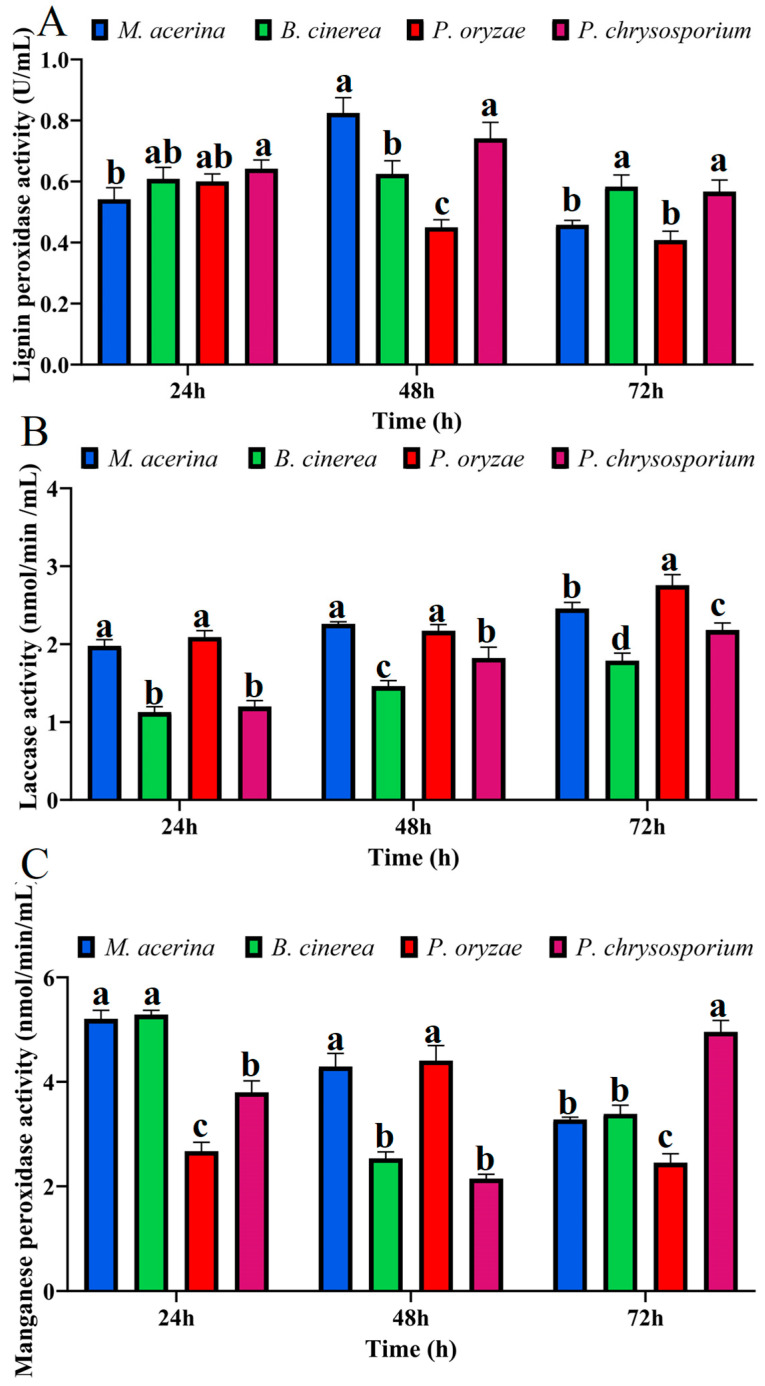
Lignin-degrading enzyme activities of four fungal species at different culture time points. (**A**) Lignin peroxidase activities. (**B**) Laccase activities. (**C**) Manganese peroxidase activities. Different letters above the plots indicate significant differences between treatments (*p* < 0.05, n = 3).

For lignin peroxidase (Figure 8A), at 24 hpi, *P. chrysosporium* showed the strongest activity, significantly higher than *M. acerina*, but not significantly different from *B. cinerea* or *P. oryzae*. At 48 hpi, *M. acerina*’s activity rose sharply, becoming not significantly different from *P. chrysosporium*, yet significantly higher than *B. cinerea* and *P. oryzae*. At 72 hpi, activities declined across all four fungi, but *B. cinerea* exhibited the highest activity, followed by *P. chrysosporium*; both were significantly higher than *M. acerina* and *P. oryzae*.

For laccase activity (Figure 8B), at 24 hpi, *M. acerina* and *P. oryzae* were both significantly higher than *B. cinerea* and *P. chrysosporium*. At 48 hpi, the pattern was similar to that at 24 hpi, except that *P. chrysosporium* was significantly higher than *B. cinerea*. At 72 hpi, *P. oryzae* continued to increase and was significantly higher than *M. acerina* and the other two fungi; *M. acerina* also increased and remained significantly higher than *B. cinerea* and *P. chrysosporium*.

For manganese peroxidase (Figure 8C), at 24 hpi, *M. acerina* and *B. cinerea* had the highest activities, significantly exceeding *P. chrysosporium* and *P. oryzae*; additionally, *P. chrysosporium* was significantly higher than *P. oryzae*. At 48 hpi, *M. acerina* and *P. oryzae* showed the highest activities, significantly higher than *B. cinerea* and *P. chrysosporium*. At 72 hpi, *P. chrysosporium*’s activity rose abruptly and became significantly higher than the other three fungi; meanwhile, *M. acerina*’s activity declined, becoming not significantly different from *B. cinerea* but remaining significantly higher than *P. oryzae*.

## 4. Discussion

This study presents the first genome sequencing of *M. acerina*, the pathogen responsible for PRSD, and provides a comprehensive analysis of the genomic features of this fungus. Taxonomic distribution analysis of sequences matched in the Nr database revealed that *M. acerina* shares an 18.86% similarity with *Phaeosphaeria nodorum*, the causal agent of wheat glume blotch—a necrotrophic fungal pathogen [43,44]. The similarity in protein composition between *M. acerina* and *P. nodorum* suggests that they may share certain functional characteristics. Additional fungal species identified through Nr database alignment include *Leptosphaeria maculans* (the causal agent of blackleg disease in oilseed rape), *Pyrenophora teres* (the pathogen of barley net blotch), and *Pyrenophora tritici-repentis* (the pathogen of wheat tan spot) (Figure S2). This indicates that *M. acerina* shares some functional resemblance with these pathogens; however, the relatively low overall similarity suggests that *M. acerina* also possesses unique functional properties. Comparative genomic analysis showed that the total genome size of *M. acerina* is larger than the average genome size of Ascomycota fungi (36.91 Mb) [45]. It is similar in size to the genomes of *B. cinerea* and *P. chrysosporium*, but smaller than those of *P. oryzae* and *E. necator*. In terms of predicted protein-coding gene numbers, *M. acerina* has fewer genes than *B. cinerea*, *P. chrysosporium*, and *P. oryzae*, but more than *E. necator*. In microbial metabolism, PKS and NRPS pathways play major roles in the synthesis of secondary metabolites, which are often closely associated with the pathogenicity of pathogenic fungi [46,47,48]. In this study, a total of 42 secondary metabolite synthesis genes were identified in *M. acerina*, a number comparable to the 44 found in *B. cinerea*, lower than the 56 in *P. oryzae*, but higher than those in *P. chrysosporium* and *E. necator*. The types of secondary metabolites are also largely similar to those of *B. cinerea*, suggesting that *M. acerina* may possess considerable pathogenic potential. Overall, the sequencing of the *M. acerina* genome, coupled with comprehensive functional prediction and comparative analysis of its protein-coding genes, provides a foundational framework for understanding the biological functions and pathogenic mechanisms of this fungus.

The plant cell wall is primarily composed of polysaccharides such as pectin, cellulose, hemicellulose, and lignin. To adapt to their own growth requirements and environmental changes, cell wall compositions vary substantially among different plant species and tissues [49]. For phytopathogenic fungi, successful infection of a host plant requires a repertoire of cell wall-degrading enzyme families adapted to the host’s specific cell wall composition. Previous studies have shown that the diversity and abundance of CAZymes are highly correlated with parasitic lifestyles [50,51]. Pathogens with distinct parasitic strategies possess different numbers and types of CAZyme families. Specifically, non-obligate parasitic fungi harbor significantly more genes involved in the degradation of plant cell wall components such as cellulose, pectin, and lignin than obligate parasites [52]. In this study, *M. acerina* was found to possess 248 genes encoding PCWDEs, ranking just below *P. oryzae* (249) but exceeding *B. cinerea* (209) and *P. chrysosporium* (174). In contrast, the obligate parasite *E. necator* contained only 15 PCWDE-encoding genes, reflecting its highly specialized mode of nutrient acquisition. Regarding the distribution of genes encoding four major categories of PCWDEs, the numbers of genes associated with cellulose, hemicellulose, and lignin degradation in *M. acerina* were comparable to those of *B. cinerea*. Moreover, compared to the other fungi, *M. acerina* exhibited significant expansion in gene families associated with AA3, PL1, and PL3 enzymes. The AA3 family comprises enzymes involved in lignin degradation, while the PL1 and PL3 families encode pectate lyases that play crucial roles in pectin degradation during pathogenesis. These findings suggest that *M. acerina* possesses a strong potential for degrading lignin and pectin. Although *P. oryzae* harbored the largest numbers of genes for cellulose, hemicellulose, and lignin degradation, it contained relatively few pectinase genes—amounting to only 23.08% of those in *M. acerina* and 41.02% of those in *B. cinerea*. As a model white-rot fungus renowned for its potent lignin-degrading capacity [53,54], *P. chrysosporium* (strain ATCC 20696) was predicted to contain 106 lignin-degrading enzyme genes in this study, slightly more than *M. acerina* (99) and *B. cinerea* (93), though the differences were modest. *P. oryzae* exhibited 117 such genes, surpassing *P. chrysosporium*, suggesting that *P. oryzae*, *B. cinerea*, and *M. acerina* possess strong potential capabilities for lignin degradation. However, both *P. chrysosporium* and *P. oryzae* contained relatively few pectinase genes, indicating weaker capacities for pectin degradation. This prediction was further supported by subsequent enzymatic activity assays.

## 5. Conclusions

In this study, *M. acerina* underwent whole-genome sequencing using Nanopore third-generation sequencing technology, achieving a sequencing depth of 128.03×. The assembled genome spans a total length of 37.03 Mb with a GC content of 47.68%. A total of 9989 genes were predicted, with an average length of 1705.5 bp and a gene density of 270 genes per megabase. Comparative genomic analysis revealed that the genome size of *M. acerina* is larger than the average genome size of Ascomycota fungi and is comparable to those of *B. cinerea* and *P. chrysosporium*. Annotation identified 42 secondary metabolite biosynthetic genes in *M. acerina*, which is close to the 44 found in *B. cinerea*, fewer than the 56 in *P. oryzae*, but more than those in *P. chrysosporium* and *E. necator*. *M. acerina* encodes 248 enzymes related to plant cell wall degradation, a number second only to the 249 found in *P. oryzae*, and higher than the 209 in *B. cinerea* and 174 in *P. chrysosporium*. Experimental assessments of PCWDEs activities in *M. acerina*, *P. oryzae*, *B. cinerea*, and *P. chrysosporium* were consistent with genomic predictions. These results confirm that *M. acerina* possesses strong abilities to degrade cellulose, hemicellulose, pectin, and lignin, which may be among the key factors contributing to its high pathogenicity toward *P. notoginseng*.

## Figures and Tables

**Table 2:** Genome features of *M. acerina*.

Statistics	*M. acerina*
Size/bp	37,037,236
Sequencing depth	128.03 X
% G + C content	47.68%
N50/bp	1,663,926
Repeat content/%	9.37%
Protein-coding genes	9989
Average gene length/bp	1705.5
Gene density	270
Exons per gene	2.8
Average exon length/bp	542.31
Introns per gene	1.8
Average intron length/bp	103.69

**Table 3:** Basic characteristics of five fungal genomes.

Genomic Information Statistics	*M. acerina*	*P. Chrysosporium* [32]	*P. oryzae* [33]	*B. cinerea* [34]	*E. necator* [35]
Size/Mb	37.04	39.21	42.70	39.50	52.51
% G + C content	47.68%	56.47%	50.09%	46.20%	39%
N50/bp	1,663,926	966,363	6,129,159	562,000	21,400
Protein-coding genes	9989	13,560	13,746	16,360	6484

**Table 4:** Comparison of CAZymes.

Species	GHs	GTs	CEs	AAs	CBMs	PLs	Total
*M. acerina*	231	80	37	109	6	36	499
*P. chrysosporium*	185	56	18	108	3	11	381
*P. oryzae*	254	95	52	132	4	7	544
*B. cinerea*	231	89	36	97	7	9	469
*E. necator*	62	52	9	11	2	0	136

## Data Availability

All data are available in Section 3 and Supplementary Materials. The genomic data of *M. acerina* are available in the NCBI database at https://www.ncbi.nlm.nih.gov/bioproject/PRJNA1347288/ (accessed on 21 October 2025) under accession number PRJNA1347288.

## Supplementary Materials

The following supporting information can be downloaded at: https://www.mdpi.com/article/10.3390/jof11110811/s1, Figure S1: Symptoms of round spot disease in *Panax notoginseng*. Figure S2: Distribution of homologous species compared in Nr database. Table S1: Pathogenicity differences of *M. acerina* isolates. Table S2: Statistics of gene function annotation results. Table S3: The core genes involved in the biosynthesis of secondary metabolites.
