# Supplementary material for: Characterization and Genome Analysis of Mycocentrospora acerina, the Causal Agent of Panax notoginseng Round Spot Disease in China
Source: J Fungi (Basel). 2025 Nov 15;11(11):811. doi: 10.3390/jof11110811 (PMC12653496; doi:10.3390/jof11110811)
Supplement: Supplementary file 1 [file jof-11-00811-s001.zip › jof-3977748-supplementary.pdf]

Supplementary Material

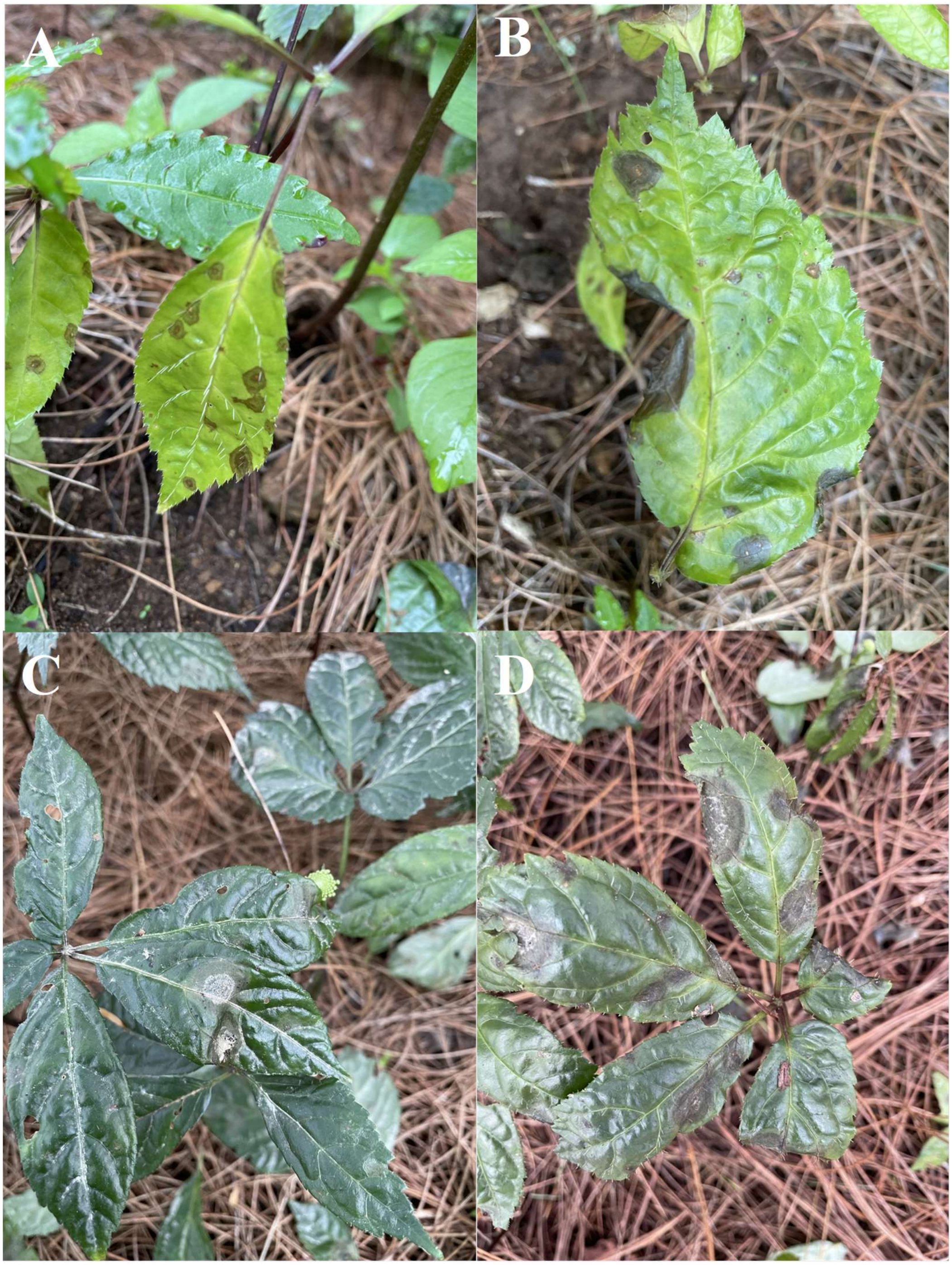

Figure S1. Symptoms of round spot disease in *Panax notoginseng*. (A) At the initial stage of the disease, small, water-soaked lesions appear on the leaves. (B-C) In the middle stage of disease development, the lesions further enlarge and darken in color, and a grayish-white fungal layer appears on the surface of the affected area. (D) In the later stages of disease development, multiple lesions fuse together, resulting in leaf wilting, decay, and abscission.

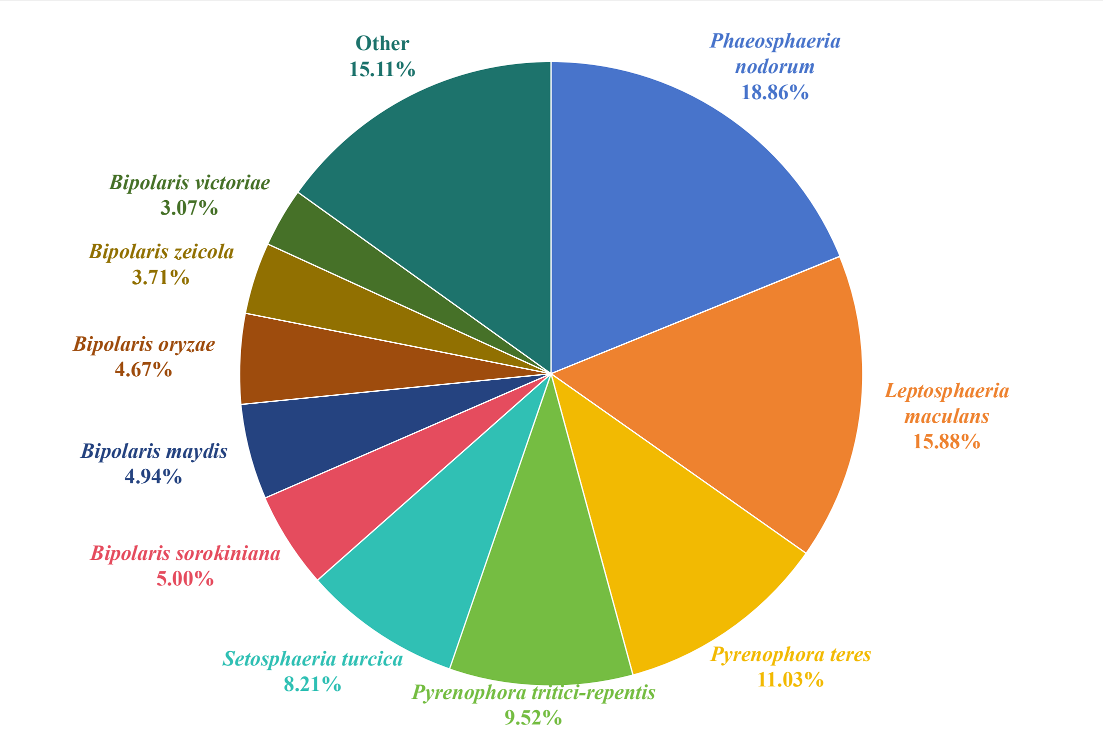

Figure S2. Distribution of homologous species compared in Nr database.

Table S1. Pathogenicity differences of *M. acerina* isolates.

Isolates	Lesion diameter（cm）	Pathogenicity	Isolates	Lesion diameter（cm）	Pathogenicity
DLanC16	1.49±0.0416	3	DLanC12	1.29±0.0681	3
DLanC19	1.49±0.0351	3	DLanC30	1.26±0.0265	3
DLanC23	1.48±0.1323	3	DLanC27	1.17±0.3051	3
DLanC29	1.46±0.0351	3	DLanC01	1.16±0.1464	3
DLanC24	1.44±0.0586	3	DLanC22	1.16±0.1007	3
DLanC03	1.44±0.1271	3	DLanC02	1.14±0.3264	3
DLanC25	1.43±0.0351	3	DLanC11	1.10±0.0693	3
DLanC18	1.42±0.0751	3	DLanC04	1.07±0.1212	3
DLanC20	1.42±0.0436	3	DLanC08	1.03±0.0785	3
DLanC21	1.41±0.0708	3	DLanC05	1.02±0.0416	3
DLanC26	1.40±0.0808	3	DLanC17	0.99±0.1653	2
DLanC15	1.40±0.0304	3	DLanC10	0.82±0.1106	2
DLanC06	1.32±0.1002	3	DLanC09	0.78±0.0747	2
DLanC28	1.30±0.0551	3	DLanC14	0.67±0.0529	2
DLanC07	1.29±0.0322	3	DLanC13	0.58±0.3696	2

Table S2. Statistics of gene function annotation results.

Database	Number	100<=length<300	length>=300
GO_Annotation	5,394	1,179	4,162
KEGG_Annotation	3,340	801	2,486
KOG_Annotation	5,461	1,143	4,270
Pfam_Annotation	7,724	1,800	5,852
Swissprot_Annotation	6,353	1,377	4,911
TrEMBL_Annotation	9,871	2,606	7,161
nr_Annotation	9,867	2,601	7,162
All_Annotated	9,877	2,608	7,165

Table S3. The core genes involved in the biosynthesis of secondary metabolites.

Species	PKS	NRPS	Terpene	Indole	Other	Total
M. acerina	16	18	8	0	0	42
P. chrysosporium	2	18	16	0	1	37
P. oryzae	23	22	11	0	0	56
B. cinerea	14	19	8	2	1	44
E. necator	0	1	4	0	0	5
